# The Effects of a Carbohydrate Hydrogel System for the Delivery of Bicarbonate Mini-Tablets on Acid–Base Buffering and Gastrointestinal Symptoms in Resting Well-trained Male Cyclists

**DOI:** 10.1186/s40798-024-00684-x

**Published:** 2024-02-15

**Authors:** Lewis A. Gough, S. Andy Sparks

**Affiliations:** 1 Human Performance and Health Research Group, Birmingham City University, Birmingham, UK; 2 Sports Performance, Exercise and Nutrition Research Group, Department of Sport and Physical Activity, Edge Hill University, Ormskirk, UK

**Keywords:** Sodium bicarbonate, Extracellular buffer, Ergogenic aid, Acid–base balance, Supplement

## Abstract

**Background:**

A new commercially available sodium bicarbonate (SB) supplement claims to limit gastrointestinal (GI) discomfort and increase extracellular buffering capacity. To date, no available data exists to substantiate such claims. Therefore, the aim of this study was to measure blood acid–base balance and GI discomfort responses following the ingestion of SB using the novel “Bicarb System” (M-SB). Twelve well-trained male cyclists completed this randomised crossover designed study. Maximal oxygen consumption was determined in visit one, whilst during visits two and three participants ingested 0.3 g∙kg^−1^ BM SB using M-SB (Maurten, Sweden) or vegetarian capsules (C-SB) in a randomised order. Finger prick capillary blood samples were measured every 30 min for pH, bicarbonate (HCO_3_^−^), and electrolytes (potassium, chloride, calcium, and sodium), for 300 min. Visual analogue scales (VAS) were used to assess GI symptoms using the same time intervals.

**Results:**

Peak HCO_3_^−^ was 0.95 mmol∙L^−1^ greater following M-SB (*p* = 0.023, *g* = 0.61), with time to peak HCO_3_^−^ achieved 38.2 min earlier (117 ± 37 vs. 156 ± 36 min; *p* = 0.026, *r* = 0.67) and remained elevated for longer (*p* = 0.043, *g* = 0.51). No differences were observed for any electrolytes between the conditions. Aggregated GI discomfort was reduced by 79 AU following M-SB (*p* < 0.001, *g* = 1.11), with M-SB reducing stomach cramps, bowel urgency, diarrhoea, belching, and stomach-ache compared to C-SB.

**Conclusions:**

This is the first study to report that M-SB can increase buffering capacity and reduce GI discomfort. This presents a major potential benefit for athletes considering SB as an ergogenic supplement as GI discomfort is almost eliminated. Future research should determine if M-SB is performance enhancing.

## Key Points

- The novel ‘Bicarb System’ (M-SB) reduced, and almost eliminated the gastrointestinal (GI) discomfort compared to vegetarian capsules (C-SB).
- The changes in acid-base balance following ingestion of M-SB were significantly greater compared to C-SB. It is unkown if this would translate to increased performance benefits, however, and the next step therefore is to determine the performance responses from M-SB.
- The increase in HCO_3_^−^ was sustained >5 mmol L^−1^ HCO_3_^−^ for longer with M-SB ingestion versus C-SB. This might suggest there is an “ergogenic window”, and ingestion timing could therefore be flexible prior to exercise.

## Background

Sodium bicarbonate (SB) is a recommended supplement to improve exercise performance, with the most recent International Olympic Committee (IOC) supporting its use in doses of 0.2–0.4 g∙kg^−1^ BM [1]. Whilst the use of SB is generally supported by peer-reviewed evidence, sports medicine practitioners, and athletes, one major drawback is the onset of gastrointestinal (GI) discomfort following ingestion that affects some athletes. Common side effects include stomach-ache, bloating, belching, diarrhoea, and in severe cases, vomiting [2]. In one severe case, Kahle et al. [3] previously reported that 10 out of 11 participants suffered from diarrhoea following ingestion of 0.3 g∙kg^−1^ BM SB dissolved in solution. As the solution form of SB seems to elicit the greatest severity of GI discomfort [4], some practitioners, athletes and researchers have opted to encapsulate the sodium bicarbonate inside digestible capsules such as gelatine or cellulose in order to try to limit the GI discomfort.

Additional attempts have also been made to find solutions to the GI problems with the use of delayed release [5] and enteric coatings of capsules and tablets [6]. These methods are designed to bypass the exposure of the exogenous bicarbonate to stomach acid, and in so doing, reduce the likelihood of bloating and other GI symptoms. These studies usually use an ingestion dose of 0.3 g∙kg^−1^ BM SB and they result in acid–base changes that are typically ergogenic (> 5 mmol∙L^−1^ blood bicarbonate, HCO_3_^−^), but GI discomfort is still present in some individuals [5]. Even with enteric coated capsules, multiple participants report moderate bowel urgency, diarrhoea, and flatulence. In recognition of these issues, further work has investigated lower doses (0.2 g∙kg^−1^ BM) of SB in solution or capsule form, however, GI symptoms are often still reported [7, 8]. Despite some successes, there are still practical limitations with supplementation, as firstly, a solution form of SB lacks palatability, and secondly, capsule ingestion requires ~ 20–25 large capsules to be ingested (for a 75 kg athlete). One further strategy to reduce the GI symptoms and allow sufficient acid–base changes to occur, is the method of ingesting SB split across multiple times in smaller doses. This is usually completed across the pre-exercise and initial exercise phases when ingesting SB [9], although this can logistically be difficult depending on the time of the event (i.e. morning events) and how easy it is to administer SB during exercise, especially for short duration events.

Based on this evidence, it is reasonable to suggest that existing strategies have failed to adequately reduce GI discomfort and to make it simultaneously practical and convenient for use in an exercise context, as this could be a barrier to ingestion. As a result, further strategies are required to reduce GI discomfort. One such strategy is a recently released commercially available SB-based product, that purports to provide potentially ergogenically significant increases in acid–base balance and is ingested just once, prior to exercise, all whilst reducing GI symptoms. This product, known as the “Bicarb System” (Maurten, Gothenburg, Sweden) uses a carbohydrate (CHO) gel to deliver SB mini-tablets that are small enough to allow passage through the pyloric sphincter, thereby, avoiding disturbance of stomach acid. Other methods, such as vegetarian capsules (C-SB) must be recycled through the antral mill prior to passing into the intestine, which subsequently leads to SB dissolving in the stomach and causing GI upset [10]. In theory, this “Bicarb System” (M-SB) removes the logistical burden of ingesting large amounts of capsules and with just one ingestion point, could make it more practical than splitting the doses. At present, it is unclear what the GI symptom or the blood acid–base balance responses are to M-SB ingestion. Therefore, the purpose of this study was to assess the acid–base balance and GI discomfort responses following the ingestion of 0.3 g∙kg^−1^ BM of SB using the M-SB compared to C-SB in well-trained cyclists.

## Methods

### Participants

Twelve well-trained cyclists [11] were recruited for this study (age: 30 ± 7 years; body mass (BM): 77 ± 5 kg; maximal rate of oxygen consumption (VO_2max_): 66 ± 5 ml∙kg^−1^∙min^−1^; peak power output (PPO) at VO_2max_: 423 ± 19 W). Weekly training was at least ≥ 3 sessions, for at least ≥ 5 h, and all had a minimum of two years training experience. No participants were ingesting other intra- or extracellular buffering substances at the time of the study. All participants provided written informed consent to take part in the study and ethical approval was granted by the institutional ethics committee (Birmingham City University approval number: #10651; date: May 2022) and the study was conducted within the ethics of the Declaration of Helsinki. Data collection was undertaken between January and July 2023, and at Birmingham City University.

### Study Design

Participants attended the laboratory on three separate occasions in a randomised, crossover (balanced Latin-square), double-blind designed study. Participants completed an initial maximal oxygen consumption test and then two trials to determine blood acid–base balance responses to ingestion of 0.3 g∙kg^−1^ BM of SB. Constraints on alcohol and caffeine were placed on participants 24 h prior to any trial, and nutrition intake was monitored and replicated for 24 h prior to each trial using the snap-n-send photographic verification method [12]. Trials were conducted at a similar time of day (± 2 h) to account for circadian variability.

### Experimental Overview

The initial visit consisted of a VO_2max_ test, using a protocol previously used with trained cyclists [13]. This was conducted to determine training status for participant characterisation. Participants completed a warm-up (10 min at 50 W) on a cycle ergometer (Lode Excalibur, Lode, Germany) and then performed graded exercise test to exhaustion, with increases of 25 W∙min^−1^. All participants kept a consistent cadence of between 85 and 95 rev∙min^−1^. Oxygen consumption (VO_2_), carbon dioxide (VCO_2_), and respiratory exchange ratio (RER) were measured continuously using a breath-by-breath gas analyser (Cortex Metalyser, Cranlea, UK). Determination of VO_2max_ was defined as the highest plateau reached, that being two successive maximal readings within 0.15 L∙min^−1^ [13]. Heart rate was recorded continuously throughout using a chest-based heart rate monitor (Polar H10, Polar, Finland) and rating of perceived of exertion (Borg 6–20; [14]) was recorded every 2 min (every 50 W stage). Finally, finger capillary blood lactate was taken pre- and post-exercise (Lactate Pro 2, Arkray, Japan). All VO_2max_ tests met the set criteria for achievement of a valid maximal test [15].

Participants then attended the laboratory on two further separate occasions. Approximately 2 h prior to the trial, participants were encouraged to ingest a 2 g∙kg^−1^ BM carbohydrate meal to mimic the practices of trained cyclists in training and competition [16]. Following dietary recall participants ingested 1.6 g^.^kg^−1^ BM carbohydrate, 18 g protein, 11 g fat, totalling 665 kcal. A fingertip capillary blood sample in a heparin-coated glass clinitube (70 μl; Radiometer Medical Ltd., Denmark) was taken for analysis of pre-ingestion acid–base status, where blood pH, bicarbonate (HCO_3_^−^), sodium (Na^+^), chloride (Cl^−^), calcium (Ca^2+^), and potassium (K^+^) were measured immediately following collection using a valid and reliable blood gas analyser (ABL9, Radiometer Medical Ltd., Denmark). Participants then ingested 0.3 g∙kg^−1^ BM SB in either vegetarian capsules (~ 1 g per capsule, size 00, Bulk Powders, UK) (C-SB) or the novel “Bicarb System” (Maurten, Sweden) (M-SB). A hydrogel carbohydrate product was also provided with both formulations of SB containing ~ 40 g of carbohydrate (Maurten, Sweden). The comparison treatment was vegetarian capsules following consultation with multiple world class athletes and sports nutritionists who highlighted this is the most common approach in practice. Following ingestion, acid–base balance responses were measured using repeated capillary blood samples every 30 min for a period of 300 min. During this time, participants were resting quietly and were permitted to ingest water ad libitum (and this was recorded). Gastrointestinal (GI) discomfort was also monitored at the same time point of blood collection using a visual analogue scale (VAS) for nausea, flatulence, stomach cramping, belching, stomach-ache, bowel urgency, diarrhoea, vomiting, thirst, and stomach bloating [17]. Each individual symptom was scored out of 10, with 10 representing “most severe symptoms” and 0 “no symptoms”. Participants were permitted to ingest water *ab libitum*, and this was replicated for each trial. Finally, at the end of the trial to complete a supplement belief questionnaire that assessed if they could detect which supplement they had ingested. This was based on a 0–10 confidence score initially (“0” = no confidence; “5” = not sure; “10” = highest confidence), followed by identification of which supplement they had. A confidence score > 5 was considered that the participant could detect which supplement they had ingested, and this was then compared against the true treatment for correct or incorrect identification.

### Statistical Analysis

All data were assessed for normality using a Shapiro Wilk test and visually inspected using boxplots. One outlier was subsequently removed from the analysis of the HCO_3_^−^ data having been identified on the boxplot via the Tukey method. Blood acid–base (HCO_3_^−^ and pH) and electrolyte (Na^+^, Ca^2+^, K^+^, Cl^−^) parameters were analysed using repeated measures analysis of variance (ANOVA) over the pre- and post-ingestion period. Post-hoc tests were conducted using Bonferroni pairwise comparisons to determine possible differences between time points within trials and between conditions at each time point, by adding this command to the syntax of the analysis. Effect sizes were calculated using partial eta squared (pη^2^). Comparisons between peak HCO_3_^−^ responses and the duration of time which HCO_3_^−^ stayed ≥ 5 mmol∙L^−1^ above the baseline value between ingestion types, were determined using paired t tests, with Hedge’s g used to calculate the effect size [18]. Gastrointestinal symptom severity totals, ratings of thirst and ad libitum fluid intake were analysed using a Wilcoxon test as were comparison of blood HCO_3_^−^ time to peak (TTP) following each ingestion strategy. Effect sizes for the Wilcoxon tests were calculated using r (where *r* = *z*/√*n*). Effect sizes were interpreted as small (0.01), medium (0.06), or large (0.14) for pη^2^, and as small (0.2), medium (0.5), or large (0.8) for g and r, as suggested by Cohen [19]. All data were analysed using SPSS (v29 for Windows, IBM Corp, Chicago, USA) and statistical significance was determined as *p* < 0.05.

## Results

**Fig. 1:**
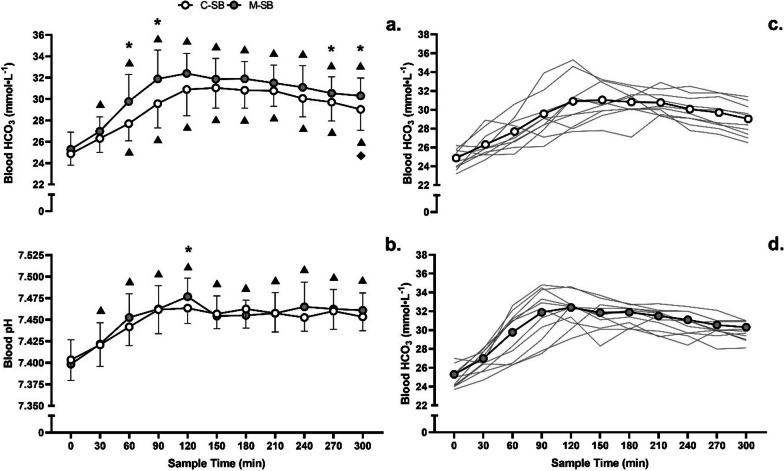
Mean (± SD) blood bicarbonate (**a**) and pH (**b**) and individual responses to sodium bicarbonate ingestion in capsules (C-SB) (**c**) and the bicarb delivery system (M-SB) (**d**). (*) denotes a significant difference between delivery methods (*p* < 0.05). (▲) denotes a significant increase from the pre-ingestion sample time (*p* < 0.05)

**Fig. 2:**
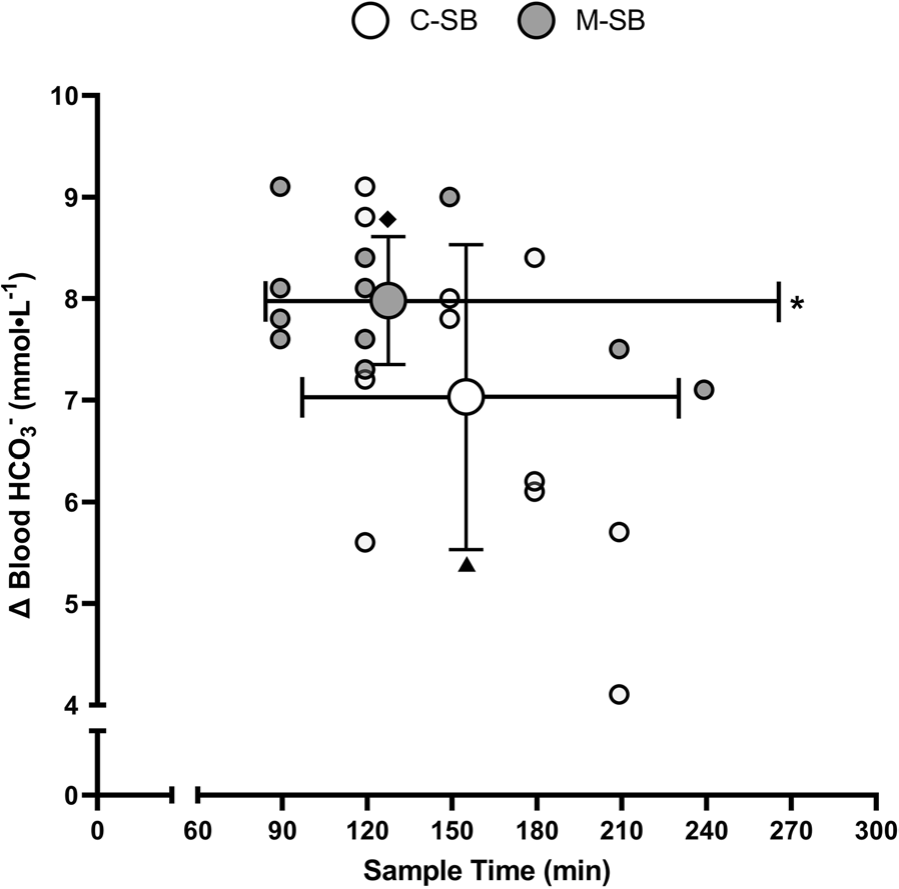
Blood bicarbonate change responses (Cmax) following sodium bicarbonate ingestion in capsules (C-SB) and the bicarb delivery system (M-SB). Large dots represent mean responses with vertical error bars representing ± SD. Horizontal error bars represent the mean duration of blood bicarbonate being > 5 mmol⋅L^−1^. (♦) denotes a significantly higher Cmax (*p* < 0.05). (*) denotes a significantly longer period of time at blood bicarbonate concentrations > 5 mmol⋅L^−1^ (*p* < 0.05). (▲) denotes a significantly longer time to peak blood bicarbonate concentration (*p* < 0.05)

Blood HCO_3_^−^ concentrations (Fig. 1a, c, d) became elevated following ingestion in both conditions (*f* = 66.18, *p* < 0.001, *pη*^2^ = 0.87), but these changes occurred to a greater extent following M-SB (*f* = 21.88, *p* = 0.001, *pη*^2^ = 0.69) in the absence of a condition*time interaction (*f* = 1.76, *p* = 0.077, *pη*^2^ = 0.15). These changes also resulted in increases in blood pH (*f* = 21.68, *p* < 0.001, *pη*^2^ = 0.66), and after 120 min, they remained elevated for the remainder of the protocol (Fig. 1b), but there was no difference in the pH responses between the ingestion conditions (*f* = 0.57, *p* = 0.466, *pη*^2^ = 0.05) nor was there a condition*time interaction (*f* = 0.97, *p* = 0.472, *pη*^2^ = 0.08). The Cmax response (Fig. 2) was highest following M-SB (mean difference = 0.95 mmol∙L^−1^, *t* = 2.25, *p* = 0.023, *g* = 0.61) and occurred more rapidly (*z* = 2.23, *p* = 0.026, *r* = 0.67) after ingestion (117.3 ± 36.6 min) than after C-SB ingestion (155.5 ± 35.8 min). Furthermore, the duration at which the change in blood HCO_3_^−^ concentrations remained > 5 mmol∙L^−1^ was also significantly longer following M-SB ingestion (mean difference = 42.5 min, *t* = 1.88, *p* = 0.043, *g* = 0.51).

**Fig. 3:**
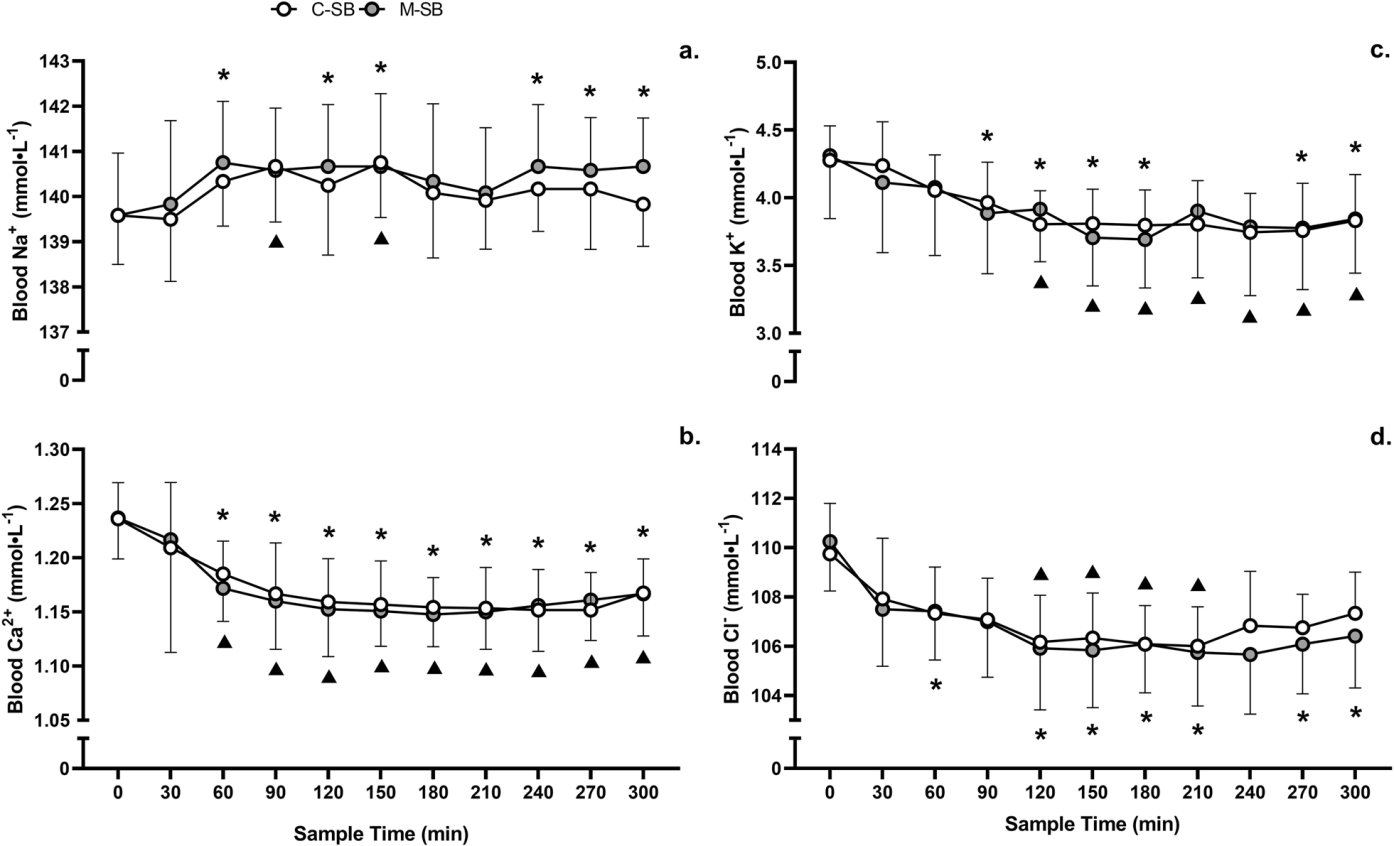
Mean (± SD) blood sodium (Na^+^) [**a**], calcium (Ca^2+^) [**b**], and potassium (K^+^) [**c**] and chloride (Cl^−^) [**d**] responses to sodium bicarbonate ingestion in capsules (C-SB) and the bicarb delivery system (M-SB). (*) denotes a significant difference in the blood concentration from the pre-ingestion sample time for M-SB (*p* < 0.05). (▲) denotes a significant difference in the blood concentration from the pre-ingestion sample time for C-SB (*p* < 0.05)

Blood Na^+^ (Fig. 3a) concentrations were the only electrolyte that increased in response to SB ingestion (*f* = 2.76, *p* = 0.041, *pη*^2^ = 0.20) but there was no main effect of condition (*f* = 1.69, *p* = 0.220, *pη*^2^ = 0.13) despite peak changes being observed at 60 min (*p* = 0.012) and 90 min (*p* = 0.03) following M-SB and C-SB ingestion, respectively, and no condition*time interaction (*f* = 0.55, *p* = 0.847, *pη*^2^ = 0.05). Blood Na^+^ remained elevated 300 min after ingestion of M-SB (*p* = 0.012), but Na^+^ had returned to pre-ingestion concentrations in C-SB. The post-ingestion responses of the other electrolytes were characterised by decreases in Ca^2+^ (*f* = 19.94, *p* < 0.001, *pη*^2^ = 0.64), K^+^ (*f* = 15.67, *p* < 0.001, *pη*^2^ = 0.59) and Cl^−^ (*f* = 10.71, *p* < 0.001, *pη*^2^ = 0.49) (Fig. 3b, c, d, respectively). There were also no observed main effects for the ingestion conditions for Ca^2+^ (*f* = 0.64, *p* = 0.442, *pη*^2^ = 0.06), K^+^ (*f* = 0.01, *p* = 0.937, *pη*^2^ = 0.001) or Cl^−^ (*f* = 1.84, *p* = 0.203, *pη*^2^ = 0.14), despite pairwise comparisons identifying minor variations in the responses (Fig. 3). Only blood Cl^−^ had returned to a concentration not significantly different to pre-ingestion following C-SB, but not following M-SB, which remained lower (*p* = 0.001). No condition*time interactions were observed for either Ca^2+^ (*f* = 0.85, *p* = 0.586, *pη*^2^ = 0.07), K + (*f* = 0.97, *p* = 0.476, *pη*^2^ = 0.05) or Cl^−^ (*f* = 0.68, *p* = 0.739, *pη*^2^ = 0.08).

**Fig. 4:**
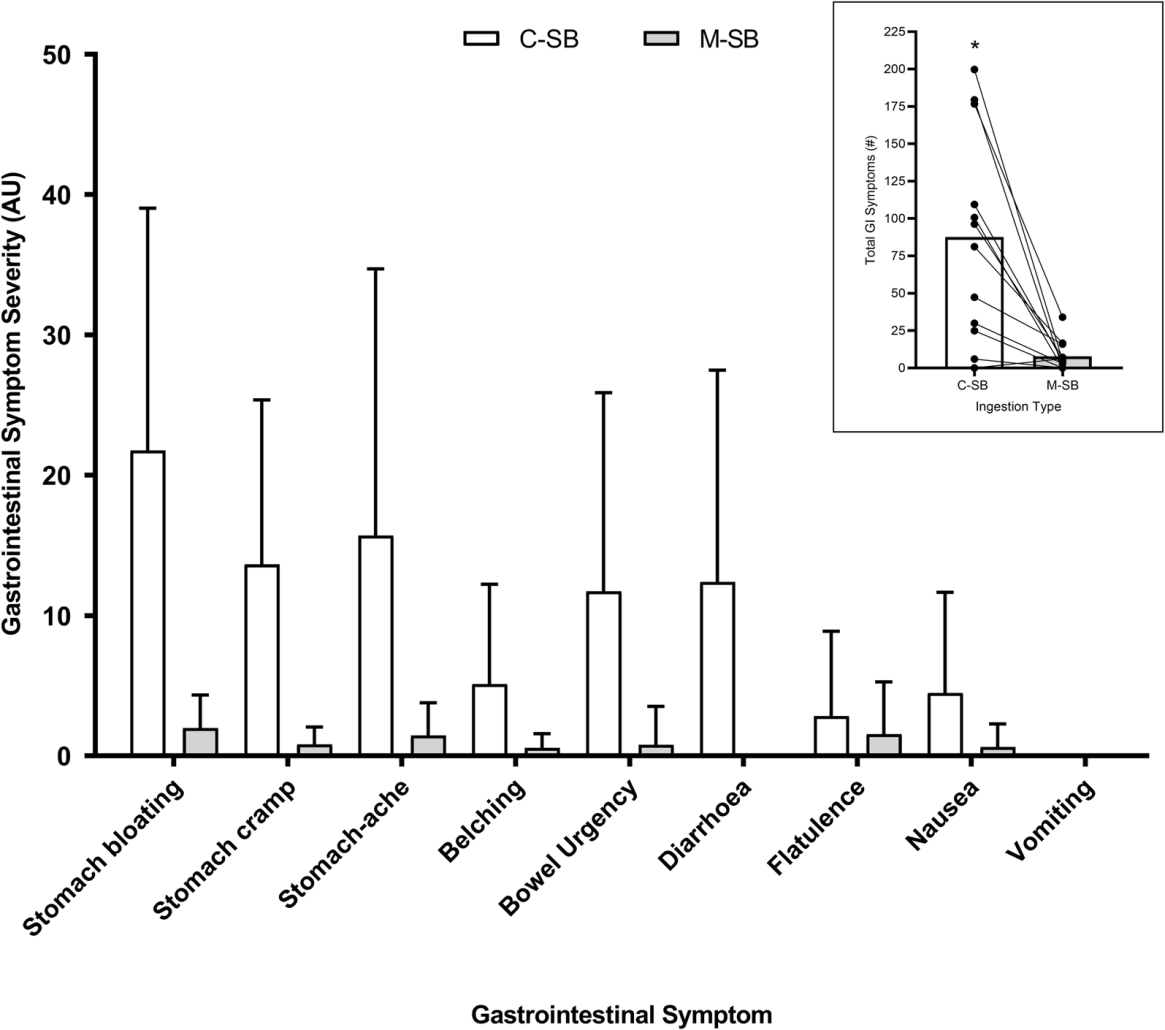
Mean (± SD) aggregated gastrointestinal symptom severity ratings for the 300 min duration of the post-ingestion period, following sodium bicarbonate ingestion in capsules (C-SB) and the bicarb delivery system (M-SB). Inserted figure represents the mean and individual total gastrointestinal (GI) responses for each ingestion type. (*) denotes a significantly higher GI symptom severity (*p* < 0.001)

**Fig. 5:**
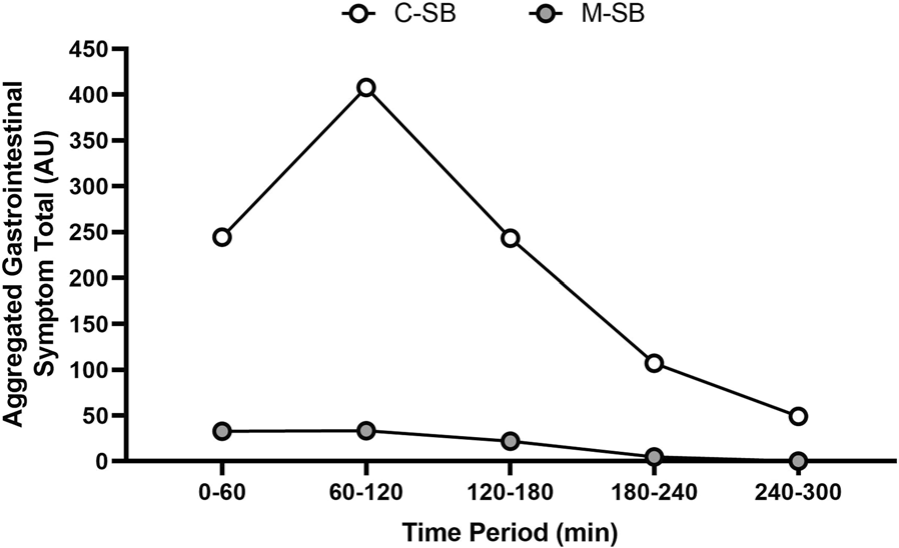
Aggregated gastrointestinal symptom total responses for each hour of the study duration, following sodium bicarbonate ingestion in capsules (C-SB) and the bicarb delivery system (M-SB)

The mean GI symptom severity responses (Fig. 4) for each symptom were greater following C-SB, and this was reflected by the significantly lower total GI symptoms reported following M-SB (*z* = −2.903, *p* = 0.004, *r* = 0.92). Diarrhoea was eliminated with M-SB despite this response being observed in C-SB, and considerably reduced all other symptoms which were observed in C-SB. Aggregated GIS totals peaked between 60 and 120 min following C-SB ingestion, but no peak was observed following M-SB ingestion (Fig. 5). The mean fluid intakes following SB administration was 1.10 ± 0.65 L and 1.04 ± 0.73 L in the C-SB and M-SB conditions respectively, but this was not affected by the method of delivery (mean difference = 0.04 L, *t* = 0.364, *p* = 0.362, *g* = 0.11). Mean thirst ratings (8.00 ± 6.84 arbitrary units (AU) and 5.42 ± 6.33 AU for C-SB and M-SB, respectively)), were unaffected by the method of delivery of the SB (mean difference = 2.58 AU, *t* = 0.516, *p* = 0.314, *g* = 0.18). Five of the participants reported headache symptoms following C-SB, but no such symptoms were reported by any participant following ingestion of in M-SB. Only one participant reporting no GI symptoms with C-SB reported a higher total GI symptom score following M-SB. For all other participants GI symptoms were lower following M-SB ingestion. Out of all participants, only one participant could detect the supplement correctly for one trial. Five trials were guessed incorrectly with the remaining trials identified as “unsure”.

## Discussion

This study aimed to assess the acid–base balance and GI discomfort response following ingestion of M-SB and C-SB in well-trained cyclists. Firstly, the findings suggest that M-SB can significantly reduce, and almost eliminate, GI discomfort compared to a more traditional method of NaHCO_3_ ingestion (i.e., C-SB). Secondly, M-SB induced a significantly greater  level of alkalosis compared to C-SB, which suggests increasing buffering capacity is possible with M-SB, although the relevance of this additional increase over C-SB in the context of exercise performance is unknown [20]. Based on this evidence M-SB may offer considerable practical benefits to athletes, as they can increase their buffering capacity sufficiently whilst avoiding the problematic GI discomfort responses typically experienced from other forms of SB ingestion. Indeed, this study reports for the first time, that M-SB appears to reduce GI discomfort to a greater extent than other previously reported GI symptom alleviating methods, including enteric coated capsules [5, 6]. Importantly, in this study a comparison with C-SB was also undertaken, consumption of which was associated with much greater frequency and severity of GI discomfort. For example, one participant suffered 9.4/10 severity for stomach cramp with C-SB, whilst in comparison, 0/10 following M-SB. Equally, another participant suffered 10/10 severity for diarrhoea, but no symptoms (0/10) with M-SB, which was also the case for three other participants.

Generally, all symptoms of GI discomfort were reduced following M-SB, but this was not the case for perceptions of thirst. This was likely due to the equimolar Na^+^ load of each supplement that was used during this study. These results support the claimed acting mechanism of absorption through the pyloric sphincter which leads to minimal disruption of stomach acid–base balance, and of CO_2_ production, resulting in near elimination of stomach related side effects. Due to the design of the present study, it is not possible to directly determine the effects of the hydrogel CHO provision on either GI symptoms or the pharmokinetic responses following administration of the SB. Provision of CHO alongside SB has previously been shown to reduce the severity of the GI side effects [4]. What is clear, is that when this hydrogel CHO product is consumed simultaneously with either M-SB or C-SB, the M-SB results in considerably reduced GI symptom severity and frequency. This product therefore has the potential to provide athletes with a practical method of ingesting a dose of potentially ergogenic SB, without the concern of it causing GI upset. What is yet to be determined, is if M-SB is also ergogenic, participants with historically severe GI symptoms from SB may now be able to improve their performance with less likelihood of upset [21]. Therefore, future studies need to investigate the efficacy of M-SB on a variety of exercise types, now it is clear that GI symptoms issues are unlikely to inhibit performance.

The increase in HCO_3_^−^ following M-SB ingestion was high enough to suggest an ergogenic effect is possible, given the Cmax was ~ 8 mmol∙L^−1^, which is far above the 5 mmol∙L^−1^ threshold suggested to be required to elicit an ergogenic effect [4, 22]. This is the first study to report this finding, and it is positive that this change has been seen from a new SB product, considering others, such as SB lotion, has no impact on any acid–base balance variable [23, 24], or enteric coated SB, which typically produces lower Cmax values [6]. The increase was also greater for a longer period of time across the 300 min testing window compared to C-SB, whereby HCO_3_^−^ was greater than 5 mmol∙L^−1^ for ~ 38 min longer, and the absolute change from baseline was ~ 1 mmol∙l^−1^ greater following M-SB ingestion. Furthermore, with HCO_3_^−^ being > 5 mmol∙L^−1^ for longer, this could suggest that a potentially ergogenic window is available following ingestion (as seen in previous vegetarian capsule data; [25]). These two interesting observations may mean that an individual time to peak approach might not be warranted. This is potentially of practical benefit to the athlete and sport medicine team, as less pressure for exact timings of peak alkalosis may be required.

It is also important to note that despite differences in HCO_3_^−^ between M-SB and C-SB, there was little difference between the electrolyte responses (Na^+^, K^+^, Cl^−^, Ca^2+^) following SB ingestion in this study. This means that if the potentially ergogenic mechanism is derived from changes in these electrolytes (or the changes these ions have on the strong ion difference) then there is likely no difference in the expected benefits to exercise performance. The very high mean increases in blood HCO_3_^−^ of almost 3 mmol∙L^−1^ over the likely ergogenic Cmax threshold of 5 mmol∙L^−1^ suggests that it may also be possible to use lower doses of SB, as these would also likely still increase HCO_3_^−^ to a sufficient level. This would also have benefits in that it would reduce the Na^+^ load with ingestion, as 0.3 g∙kg^−1^ BM is far above the recommended daily amount and SB is commonly loaded on both consecutive day and even multiple times per day (such as a competition weekend).

It is important to note that this experimental study was conducted in controlled laboratory conditions in the absence of any exercise, and therefore has some important limitations. It is likely that in a competition setting, for example, that factors such as the warm-up/preparation phase and potential changes in anxiety could impact either the blood or GI discomfort response [26]. In this initial study it was important, however, to determine the blood acid–base balance changes following M-SB given that the individual time to peak HCO_3_^−^ could be an important factor to improve the ergogenic effect [27, 28]. This is an important first step in determining the pharmokinetics of the M-SB ingestion strategy and doing so in a laboratory setting, allowed a greater level of control than in a field setting. Now this study has been completed and the acid–base responses to M-SB have been determined, it is important that future studies investigate the effect of this strategy on exercise performance and the effects of ecologically valid pre-exercise routines such as warm-ups and environmental factors (e.g. racing, heat, hypoxia). This is to ascertain if the responses are similar to those exhibited using other more well-established ingestion strategies. .

## Conclusions

This study reports that a novel SB supplement (M-SB) induces acid–base balance responses that are similar to traditional methods of SB (C-SB), whilst almost eliminating GI symptoms. With the near elimination of GI discomfort, athletes can now be confident that they can ingest M-SB prior to exercise without the deleterious impact of GI discomfort, and in a manner that only requires one intake to induce blood alkalosis. Further research is now needed to directly quantify the potentially ergogenic effects of this new supplement ingestion strategy on a variety of exercise types.

## Data Availability

The datasets used and/or analysed during the current study are available from the corresponding author on reasonable request.

## Abbreviations

SB: Sodium bicarbonate
GI: Gastrointestinal discomfort
IOC: International Olympic Committee
HCO_3_^−^: Blood bicarbonate
M-SB: ‘Bicarb system’
C-SB: Vegetarian capsules sodium bicarbonate
CHO: Carbohydrate
VO_2max_: Maximal rate of oxygen consumption
PPO: Peak power output
VO_2_: Oxygen consumption
VCO_2_: Carbon dioxide
RER: Respiratory exchange ratio
Na^+^: Sodium
Cl^−^: Chloride
Ca^2+^: Calcium
K^+^: Potassium
VAS: Visual analogue scale
ANOVA: Analysis of variance
pη^2^: Partial eta squared
C_max_: Change responses
BM: Body mass
AU: Arbitrary units
